# Hereditary pancreatic cancer: related syndromes and clinical perspective

**DOI:** 10.1186/s13053-017-0069-6

**Published:** 2017-06-28

**Authors:** Sergio Carrera, Aintzane Sancho, Eider Azkona, Josune Azkuna, Guillermo Lopez-Vivanco

**Affiliations:** 1 Hereditary Cancer Genetic Counseling Unit- Medical Oncology Department, Cruces University Hospital, Plaza de Cruces s/n. 48903, Baracaldo, Bizkaia Spain; 2 Medical Oncology Department, Cruces University Hospital, Baracaldo, Spain

**Keywords:** Pancreatic ductal adenocarcinoma, Pancreatic neuroendocrine tumor, Familial pancreatic cancer, Genetic testing, Hereditary cancer

## Abstract

Pancreatic cancer is a very aggressive disease with a poor prognosis. The majority of them are attributed to sporadic causes, especially to many modifiable risk factors such as tobacco or alcohol abuse. The principal histologic subtype of pancreatic cancer is ductal adenocarcinoma. Pancreatic neuroendocrine tumors, which constitute a more indolent entity, represent second type of pancreatic cancer in terms of incidence. Individuals with a family history of pancreatic cancer carry an increased risk of developing the disease, which may be related to an underlying hereditary component. Unfortunately, in the majority of these families the suspected germline genetic cause responsible of the disease will not be identified, but approximately in a 20% of the cases a hereditary cancer predisposition syndrome with increased risk of pancreatic cancer development can be recognized. This review will be focused on the leading hereditary cancer syndromes related to pancreatic ductal adenocarcinoma and pancreatic neuroendocrine tumors. Additionally, we will try to explain clinical aspects related to the identification of germline mutations in pancreatic cancer patients and their potential implications in oncologic treatment decisions.

## Background

From a histological point of view [1], almost 95% of pancreatic cancers are pancreatic ductal adenocarcinomas (PDAC). PDAC has the worst prognosis among the major cancers and it constitutes the fourth leading cause of cancer death in the developed world. Such is its relevance that PDAC will be the third cause of cancer mortality in European Union in 2017, with 43,800 expected deaths among women and 43.600 among men [2]. In the United States the prospects also are not looking very hopeful, with 53,670 new cases and 43,090 deaths due to pancreatic cancer predicted this year [3]. The average lifetime risk of pancreatic cancer for both men and women is about 1 in 65 (1.5%) and the five year survival rate is 7% [4], which is related to advanced stage at diagnosis in the majority of the cases.

Pancreatic neuroendocrine tumors (PNETs) are infrequent neoplasms that represent approximately 1-2% of all pancreatic cancers [5]. Estimated incidence for PNETs is less than 1/100,000 per year although their relative indolent nature could underestimate these numbers [5, 6]. Five year survival rate in PNETs is about 42%, which is in concordance with predominant diagnosis of low-intermediate grade tumors [7].

Older age constitutes one of the main risk factors in the development of PDAC. Median age of onset of PDAC is 71 years [8]. Tobacco is the most important recognized toxic risk factor, doubling the risk [9]. Alcohol abuse [10], chronic pancreatitis [11], dietary factors, obesity, exposure to different agents and Diabetes Mellitus type 2 (DM2) also increase risk for PDAC [12]. Apart from these factors, family history also can influence in the probability of PDAC development [13]. Different reports that have evaluated incidence of pancreatic cancer in relatives have found that first degree relatives have almost a two-fold increased risk of developing PDAC and also that this risk seems to be proportional to the number of first-degree relatives with PDAC [14, 15]. It is estimated that a hereditary component may be implicated in nearly 10% of all PDAC cases [16, 17], but currently in less than 20% of them a defined hereditary cancer predisposition syndrome with increased risk of PDAC development can be identified.

Conversely, in PNETs, there are no well recognized modifiable risk factors [18]. The majority of them are considered sporadic tumors and in about 20% of the cases a cancer hereditary syndrome can be recognized [19].

## Hereditary cancer syndromes related to PNETs

Main hereditary cancer syndromes related to an increased risk in PNETs development are multiple endocrine neoplasia type 1 (MEN1), Von-Hippel-Lindau disease (VHL) and neurofibromatosis type 1 (NF-1). Although less frequently, a relation between PNETs and Tuberous sclerosis complex (TSC) has been suggested. Predominantly PNETs in hereditary syndromes are grade 1 (PNET G1, Ki-67 index <3%) and grade 2 (PNET G2, Ki-67 index 3-20%) tumors [20].

MEN1, which is also referred as Wermer syndrome [21], is clinically characterized by the classical triad of tumors of the parathyroid glands, the pancreatic islet cells, and the anterior pituitary and it is inherited in an autosomal dominant manner with high penetrance. *MEN1* gene is on chromosome 11q13 and encodes menin protein [22]. Parathyroid tumors, resulting in primary hyperparathyroidism are the most common feature of MEN1 and occur in approximately 95% of patients [23]. PNETs occur in 20-75% and anterior pituitary tumors occur in 30% of patients [24]. Multifocality is one of the main features of PNETs in this syndrome [25]. Most of PNETs are non-functioning tumors [26], and insulinomas are the second group in frequency order. More rarely, glucagonomas and vipomas have been described among MEN1 related PNETs [27].

VHL disease is an autosomal dominant condition secondary to mutations in *VHL* gene, and it is typically associated with pheochromocytomas, renal cell carcinomas, central nervous system hemangioblastomas, endolymphatyc sac tumors and cystic pancreatic lesions [28]. Most of these pancreatic cysts are considered benign in a sense that they do not alter pancreatic function or they do not have an increased risk of malignancy towards PDAC. Presence of PNETs has been described in around 5-17% of patients with VHL disease, and they are almost always single non-functioning tumors [29].

NF type 1 is a disorder inherited in an autosomal dominant manner and it is caused by mutations in *NF1* gene [30]. Neurofibromas, café-au-lait spots, Lisch nodules and freckles in the underarms are typical features of this syndrome, and they are included in well defined clinical diagnosis criteria [31]. PNETs have been described in less than 1% of patients with NF type 1, with somatostatin and insulin secreting tumors being the most commonly associated lesions [32, 33]. Data about increased risk of PDAC and NF type 1 are scarce and inconclusive, with isolated cases reported in the literature [34].

TSC is a rare entity inherited in an autosomal dominant manner, characterized mainly by multiple hamartomatous lesions, epilepsy and intellectual disability, and it is produced by mutations in *TSC1* and *TSC2* genes [35]. Although there is scarcity of data about TSC and increased risk of PNETs, insulinomas and non-functioning tumors have been reported in patients with TSC [36].

## Hereditary cancer syndromes related to increased risk of PDAC

The more remarkable hereditary cancer predisposition syndromes with increased risk of PDAC are: hereditary breast and ovarian cancer syndrome (HBOC), familial melanoma (FM), Lynch syndrome (LS), familial adenomatous polyposis (FAP), Peutz-Jeghers syndrome (PJS) and Li-Fraumeni syndrome (LFS) [37]. Also *ATM* gene has been defined as a potential predisposing factor of PDAC in heterozygous carriers [38]. They account for almost 10-15% of PDAC familial cases, defined by a minimum of two PDAC diagnoses among first degree relatives [39]. The remaining 85-90% of familial cases with pancreatic cancer aggregation lacks these hereditary cancer predisposition cancer syndromes [40] and they are defined as familial pancreatic cancer (FPC). Considering that in the majority of families with PDAC susceptibility a responsible gene mutation will not be identified, several multigene panel and/or whole genome sequencing studies have been designed [41]. Multiple gene panels for PDAC include among others, *BRCA1*, *BRCA2*, *PALB2*, *CDKN2A*, *MLH1*, *MSH2*, *MSH6*, *PMS2*, *EPCAM*, *ATM*, *APC*, *STK11*, *PRSS1* and *TP53* genes [42]. A PACGENE Consortium study [43] included samples from 727 unrelated probands with PDAC family history (521 met FPC criteria) who were tested for mutations in *BRCA1*, *BRCA2*, *PALB2* and *CDKN2A*. They found that the prevalence of pathogenic variants among included probands was: *BRCA1*, 1.2%; *BRCA2*, 3.7%; *PALB2*, 0.6% and *CDKN2A* 2.5%. As a consequence of this approach, many families that meet classical definition of FPC are now being reclassified into specific hereditary cancer syndromes. Therefore, we could affirm that FPC is currently a diagnosis of exclusion, strictly reserved for those families with 2 or more first-degree relatives with PDAC in the absence of a recognizable syndrome or genetic disorder. Mean age of onset of pancreatic neoplasia in FPC seems to be slightly lower (64-65 years) than sporadic pancreatic cancer cases [44].

### Hereditary Breast and Ovarian Cancer syndrome

HBOC syndrome is caused mainly by germline mutations in *BRCA1/2* genes. *BRCA1* or *BRCA2* mutations increase risk of developing breast and ovarian cancer and they are inherited in an autosomal dominant manner. *BRCA1* or *BRCA2* mutations may be suspected in those families with multiple breast-ovarian cancer cases, especially when they are diagnosed at early age [45]. The estimated prevalence of *BRCA1* and *BRCA2* mutations is about 1/550 [46], although this number may vary depending on the selected population [47]. Data about prevalence of PDAC among *BRCA* mutations carriers are heterogeneous, but a study which performed *BRCA* testing on an unselected collected cohort of 306 patients showed that 4.6% of them had pathogenic *BRCA1* or *BRCA2* germline variants [48]. Although *BRCA1* mutations and risk of PDAC development is debatable, with studies that have shown no risk and others with a relative risk of 2.8 [49, 50], there is consensus about *BRCA2* mutations and its relationship with increased risk of PDAC, with a relative risk in a range of 2.3-7 across different published studies [48, 51]. *BRCA2* gene mutations constitute the most frequent inherited risk factor for PDAC [52].

*PALB2* (Partner and Localizer of *BRCA2*) gene mutations increase relative risk of breast cancer [53] and also it has been identified as a PDAC susceptibility gene [54]. The prevalence of *PALB2* among families with PDAC aggregation is estimated in a range from 3% to 4% [55]. PDAC relative risk associated with *PALB2* mutations is not well defined at this time [56]. Germline testing for *PALB2* gene should be considered in individuals with striking family history of breast and pancreatic cancers who have non-informative results for mutations in *BRCA1/2.* A current report yielded a *PALB2* mutation frequency of 0.05% among general population [57].

### Familial Melanoma

FM is defined by the presence of two cases of invasive melanoma among first degree relatives (*rule of two*). In geographical areas where melanoma prevalence is higher, three cases among close relatives are necessary to meet the clinical definition (*rule of three*) [58]. *CDKN2A* gene germline mutations constitute the main hereditary cause in familial melanoma, although other genes such as *CDK4* and *BAP1* have been associated to this syndrome [59]. *CDKN2A* mutations are inherited in an autosomal dominant manner [60]. Estimated prevalence of *CDKN2A* mutations among general population is 0.01% [61]. An increased risk of pancreatic cancer in FM kindred with a known *CDKN2A* mutation has widely been documented [62–64], especially in those with a specific 19 base pair *p16* pathogenic variant, referred to as *p16*-Leiden [65]. Retrospective analysis in this founder mutation group estimated a cumulative risk of 17% in suffering from pancreatic cancer [66]. Recently, a new *CDKN2A* pathogenic variant, p.D84V (c.251A > T) has been described in an Italian study which included patients with multiple primary cutaneous melanomas or with primary cutaneous melanoma associated with family history of melanoma and/or PDAC [67].

### Lynch Syndrome

LS (hereditary nonpolyposis colorectal cancer) is caused by germline mutations in mismatch repair (MMR) genes (*MLH1*, *MSH2*, *MSH6*, and *PMS2*) and *EPCAM* gene. One of the hallmarks of tumors in LS is high microsatellite instability. It represents the most common cause of hereditary colorectal cancer. Other cancer risks includes: endometrial cancer, ovarian cancer, gastric cancer, urothelial cancer, skin cancer, brain cancer and PDAC [68]. Population prevalence of LS is estimated at 1:440 [69]. There is a near 9-fold increase in risk of developing pancreatic cancer among families with pathogenic MMR gene variants compared to the general population [70]. Medullary carcinoma of the pancreas is an infrequent type of pancreatic adenocarcinoma which has been suggested to be related to LS [71].

### Familial Adenomatous Polyposis

FAP syndrome is an autosomal dominant entity characterized by hundreds to thousands of adenomas throughout the colon and also a variety of common signs, such as polyps of the gastric fundus and duodenum, osteomas, dental anomalies, congenital hypertrophy of the retinal pigment epithelium (CHRPE), thyroid, brain and periampullary carcinomas, and desmoids tumors. Individuals with FAP have a risk in developing colorectal carcinoma by the fourth decade of life that is near 100% [72]. FAP syndrome is caused by mutations in the *APC* gene [73]. Estimated prevalence of FAP among general population is over 1/10,000 [74]. In patients with FAP, the relative risk for PDAC is estimated to be 4.5 times higher than for the general population [75].

### Peutz-Jeghers syndrome

PJ syndrome is a rare and autosomal dominant entity caused by mutations in the *STK11* (serine/threonine kinase 11) */LKB1* gene. This disorder is characterized by mucocutaneous pigmentation, typically in oral mucosa and around the lips, and pathognomonic intestinal hamartomatous polyps [76]. PJ syndrome patients have an increased risk for cancers of the colon, stomach, small intestine, pancreas, breast, and other organs [77]. Prevalence of PJ is estimated from 1 in 8300 to 1 in 280 000 individuals [78]. Relative risk for PDAC in PJ syndrome is the highest of all known hereditary cancer predisposition syndromes, being estimated as high as 132 [79]. A study about cancer risk in PJ syndrome published in 2006 [80] yielded that the cumulative lifetime risk of pancreatic cancer for PJ patients was 11%. A Dutch study [81] including 144 PJ syndrome patients showed a cumulative risk for PDAC of 26% at the age of 70 and a relative risk of 76.

### Li-Fraumeni syndrome

LF syndrome is an autosomal dominant cancer predisposition condition characterized by the development of a wide spectrum of childhood and adult-onset malignancies and it is caused by germline mutations in *TP53* gene [82]. It is estimated that around 50% of the individuals with LFS will develop cancer by the age of 30 years [83]. The core cancers associated with LFS are breast cancer, sarcomas, brain tumors, adrenocortical carcinomas and leukemia. Individuals with this syndrome have also increased risk of suffering from lymphoma, melanoma, lung, PDAC, prostate and ovarian cancers [84]. Estimated prevalence of *TP53* gene mutations among general population is 0.005-0.01% [85]. The relative risk for developing PDAC is near 7-fold increased [86].

### ATM gene

*ATM* (ataxia telangiectasia mutated) gene causes ataxia telangiectasia syndrome when biallelic pathogenic variants are inherited [87]. The reported monoallelic carrier frequency of pathogenic *ATM* variants in the population is 0.5–1% [88]. Recent studies have suggested a plausible relationship between *ATM* heterozygous status and an increased risk in PDAC development. Some authors have published a 2-3 fold increased risk for PDAC [89, 90], whereas others have not found this effect [91]. A study which included 166 familial pancreatic cancer probands showed that 2.4% (4/166) carried pathogenic *ATM* variants [92]. Other study has supported this association between *ATM* heterozygous pathogenic variants carriers and PDAC risk [38].

### Hereditary pancreatitis

Repeated pancreatic injury can lead to chronic pancreatitis, increasing risk of malignant transformation. Hereditary pancreatitis (HP) is an extremely rare condition with an estimated prevalence of 3 in 1,000,000 people [93]. HP patients have 50 to 70 times relative risk for PDAC compared with general population [94] and they usually develop PDAC about 20 years earlier [95]. It is estimated that 30% to 40% of HP affected individuals will develop PDAC by the age of 70 [94]. *PRSS1* (protease, serine 1) gene, which encodes cationic trypsinogen, is the main gene related to HP. In fact, it is calculated that near 80% of patients with HP have pathogenic variants in *PRSS1* [96]. *PRSS1* gene mutations are inherited in an autosomal dominant manner, generating a scenario where activated trypsin cannot be degraded and/or activation of trypsinogen into trypsin is stimulated, leading to inflammation and pancreas self-destruction [97]. *SPINK1* (serine peptidase inhibitor, Kazal type 1) gene, which encodes a trypsin inhibitor that is secreted by the pancreatic acinar cells, is also related to HP [98]. Since the majority of *SPINK1* mutations are inherited in an autosomal recessive pattern, some of them may be inherited in an autosomal dominant manner [99]. Besides, we must consider cystic fibrosis, caused by mutations in *CFTR* gene, in differential diagnosis of HP. It is estimated that 1.5% of all patients with cystic fibrosis will suffer from pancreatitis, potentially increasing risk of PDAC development [100, 101].

**Fig. 1:**
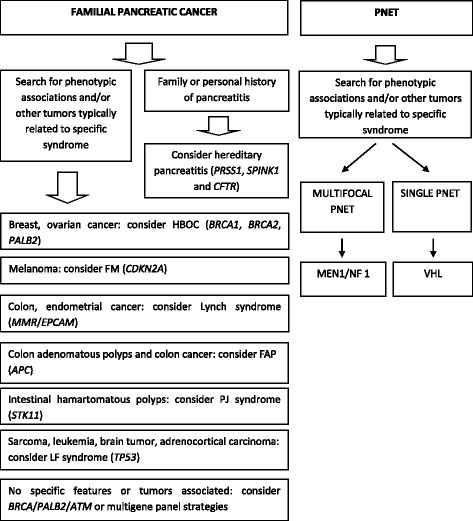
Proposed algorithm in differential diagnosis of pancreatic tumors (PDACs and PNETs) in a gene by gene strategy

An algorithm with an approximation to differential diagnosis of pancreatic tumors in familial or individuals with suspected inherited/germline component is shown in Fig. 1.

## Translational oncology: germline genetic testing in pancreatic cancer and potential impact on treatment decisions

Metastatic PDAC patients are usually treated with chemotherapy [102]. Current options in patients with good performance status are FOLFIRINOX (a platinum containing regimen) or gemcitabine/nab-paclitaxel in first line setting [103, 104] and treatment decision depends on patients’ comorbidities or expected toxicities profiles. Tumors harboring somatic or germline pathogenic variants in genes related to DNA double strand damage repair, such as *BRCA1*, *BRCA2*, *PALB2* or *ATM,* have been associated to better responses to platinum-based chemotherapy schedules [105]. Platinum compounds generate double DNA strand breaks that cannot be repaired when homologous recombination related genes are affected [106]. This benefit in platinum based schedules has also been reported in patients with PDACs related to HBOC syndrome and constitutes the basis for tailored therapy clinical trials [107–109].

Poly-ADP ribose polymerases (PARP) are involved in single DNA strand break repair. Those tumors harboring homologous recombination genes mutations can draw upon this salvage pathway in order to repair DNA damage [110]. Therefore, inhibition of PARP mediated pathway could lead to tumor cell destruction (synthetic lethality concept) in the presence of a pathogenic *BRCA* variant. Olaparib was the first PARP inhibitor approved as maintenance therapy in advanced high grade serous ovarian carcinomas that have platinum sensitive recurrences [111]. Olaparib and its copartners (veliparib, rucaparib) alone or in combination with platinum based chemotherapy have shown high activity in BRCA/PALB2 mutated pancreatic cancers and they are object of study in current open randomized clinical trials [108, 112–114].

**Fig. 2:**
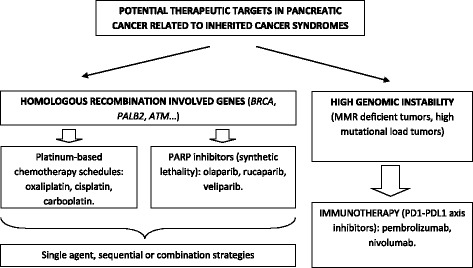
Approximation to customized PDAC treatment in the context of hereditary cancer syndromes

It is hypothesized that tumors with high genomic instability may benefit more from immunotherapy checkpoint inhibitors, especially from the program death 1/program death-ligand 1 (PD1/PD-L1) axis agents (nivolumab, pembrolizumab) [115]. Tumors harboring high genomic instability are related to a higher mutational load which potentially can increase the number of neoepitopes that are exposed and virtually generate a specific anticancer immune response [116]. Genomic instability is one of the main characteristic of Lynch syndrome related tumors and it has also been correlated with tumors with somatic alterations in *BRCA* and *PALB2* genes [117]. It has to be emphasized that a phase II study of pembrolizumab in patients with colorectal cancer showed no tumor responses in the group with mismatch repair proficient tumors, and an impressive 50% of objective responses in patients with mismatch repair deficient tumors [118]. Also, PD-1 inhibition treatment approximation has shown promising results in a phase II study including patients with different gastrointestinal cancers: those with mismatch repair deficient non colorectal tumors had an immune-related objective response rate of 71%. This fact should be taken into consideration when designing clinical trials of immunotherapy in pancreatic cancer and also in treatment decision in patients with metastatic PDACs related to hereditary cancer syndromes [119]. Figure 2 summarizes these features of PDAC in the context of hereditary cancer syndromes and their potential implication in targeted therapies development.

The majority of neuroendocrine tumors have somatic mutations in *MEN1*, *ATRX*, *DAXX* and/or in genes involved in phosphoinositide 3-kinase, AKT, and mammalian target of rapamycin (PI3K/AKT/mTOR) pathway [120, 121]. The presence of specific somatic or germline mutations in PNETs and their correlation with better responses to different multitargeted inhibitors is object of research. This way is being explored in a prospective phase II trial [122], which is recruiting patients diagnosed of low-intermediate grade neuroendocrine tumors; patients with germline/somatic *MEN1* mutations are assigned to sunitinib (multityrosine kinase inhibitor) treatment and those with germline/somatic *NF1/VHL/TSC* mutations are treated with everolimus (mTOR pathway inhibitor).

## Conclusions

PDAC is the more frequent histological subtype of pancreatic cancer. Even though the majority of pancreatic cancer cases are considered as sporadic, it is estimated that about 10% of them have a familial component. FPC is defined as a family with 2 or more first-degree relatives with pancreatic cancer. The majority of families with multiple cases of pancreatic cancer do not have an identifiable causative gene or syndrome and strictly they meet FPC definition. A small subset of hereditary pancreatic cancer (20%) is attributable to known inherited cancer predisposition syndromes. PNETs are the second type of pancreatic cancer in terms of incidence and about 20% of them have an involved inherited condition. Recognition of germline mutations in patients with pancreatic tumors does not only suppose an impact in genetic counseling process, since it also may affect treatment decisions and predict response to specific therapies.
